# The Association between Body Mass Index and Intra-Cortical Myelin: Findings from the Human Connectome Project

**DOI:** 10.3390/nu13093221

**Published:** 2021-09-16

**Authors:** Debo Dong, Yulin Wang, Zhiliang Long, Todd Jackson, Xuebin Chang, Feng Zhou, Hong Chen

**Affiliations:** 1 Key Laboratory of Cognition and Personality, Southwest University (SWU), Ministry of Education, Chongqing 400715, China; debo.dong@gmail.com (D.D.); yulin.wang90.swu@gmail.com (Y.W.); longzhiliang@swu.edu.cn (Z.L.); 2 Faculty of Psychology, Southwest University (SWU), Chongqing 400715, China; 3 Department of Psychology, University of Macau, Taipa 999078, China; tjackson173@hotmail.com; 4 School of Mathematics and Statistics, Xi’an Jiaotong University, Xi’an 710049, China; xuebin_chang@163.com; 5 Center for Information in Medicine, MOE Key Lab for Neuroinformation, The Clinical Hospital of Chengdu Brain Science Institute, School of Life Science and Technology, University of Electronic Science and Technology of China, Chengdu 610054, China; zhou.feng@live.com

**Keywords:** obesity, body mass index, myelin, cerebral cortex, T1-w/T2-w ratio, reward

## Abstract

Intra-cortical myelin is a myelinated part of the cerebral cortex that is responsible for the spread and synchronization of neuronal activity in the cortex. Recent animal studies have established a link between obesity and impaired oligodendrocyte maturation vis-à-vis cells that produce and maintain myelin; however, the association between obesity and intra-cortical myelination remains to be established. To investigate the effects of obesity on intra-cortical myelin in living humans, we employed a large, demographically well-characterized sample of healthy young adults drawn from the Human Connectome Project (*n* = 1066). Intra-cortical myelin was assessed using a novel T1-w/T2-w ratio method. Linear regression analysis was used to investigate the association between body mass index (BMI), an indicator of obesity, and intra-cortical myelination, adjusting for covariates of no interest. We observed BMI was related to lower intra-cortical myelination in regions previously identified to be involved in reward processing (i.e., medial orbitofrontal cortex, rostral anterior cingulate cortex), attention (i.e., visual cortex, inferior/middle temporal gyrus), and salience detection (i.e., insula, supramarginal gyrus) in response to viewing food cues (corrected *p* < 0.05). In addition, higher BMIs were associated with more intra-cortical myelination in regions associated with somatosensory processing (i.e., the somatosensory network) and inhibitory control (i.e., lateral inferior frontal gyrus, frontal pole). These findings were also replicated after controlling for key potential confounding factors including total intracranial volume, substance use, and fluid intelligence. Findings suggested that altered intra-cortical myelination may represent a novel microstructure-level substrate underlying prior abnormal obesity-related brain neural activity, and lays a foundation for future investigations designed to evaluate how living habits, such as dietary habit and physical activity, affect intra-cortical myelination.

## 1. Introduction

The abundance of easily accessible, high-energy palatable food has contributed to a change in how people relate to food, and is most commonly manifested as an increase in obesity [1]. According to the World Health Organization (2016), more than 1.9 billion adults are overweight, among whom 650 million are obese, with morbidity rates continuing to increase. Obesity is considered as the most significant health concern of the twenty-first century, since it substantially increases risk for a myriad of illnesses including type 2 diabetes mellitus, hypertension, elevated cholesterol, stroke, dementia, depression, obstructive sleep apnea, and several cancers, thereby contributing to declines in both quality of life and life expectancy. Unfortunately, efforts to reverse obesity have been largely unsuccessful, in part, because our understanding of its neurobiological correlates continues to be limited [2].

Previous studies investigating the association between food-related cue-induced brain activity and obesity have consistently found abnormal neural activity in an assumed network of brain reward processing areas, including the ventral striatum and orbitofrontal cortex (OFC) regions [3,4,5,6,7] as well as areas involved in visual attention and salience detection (e.g., occipital lobe, insula, supramarginal gyrus, precuneus, middle/inferior temporal gyrus) [7,8,9,10], and somatosensory processing (e.g., somatosensory cortex) [4,6,8]. However, in terms of underlying micro-structural bases of obesity, it remains unclear whether abnormal neural activities in these regions are dependent on defects or changes in levels of myelin.

Myelin is crucial for the proper function of the brain. It is an electrically insulating structure that surrounds axonal fibers, hence facilitating faster axonal signal propagation and increased signal integrity. Myelination determines the arrival time of signals from multiple axons, and therefore also influences neuronal spiking [11]. Myelinated axons are widely distributed throughout the brain but are located mainly in deep white matter. Myelinated axons, also located in cerebral cortex gray matter, are called intra-cortical myelin. Intra-cortical myelin is of great importance for the integrity of neural connections and neural synchrony of the cerebral cortex [12,13,14,15]. In humans, direct evidence suggests intra-cortical myelin is involved in cognitive functioning through its relationship with neural activity, potentially by the facilitation of local neural synchrony [16]. One previous study found even very subtle defects in myelination have a persistent impact on cortical network functions [17]. In addition, intra-cortical myelin is a sensitive indicator to track the development of neuronal activity throughout the lifespan, thus actively engaged in brain remodeling and plasticity [13,15,18]. Together, these findings suggest that intra-cortical myelination is essential for fine-tuning cortical functional circuits.

Myelin is produced and maintained via oligodendrocyte metabolism in the central nervous system [11]. Recent animal studies have established a link between obesity and impaired oligodendrocyte maturation regarding cells that produce and maintain myelin. Specifically, these animal studies have shown that: (1) high-fat diet consumption can lead to oligodendrocyte loss and trigger myelin damage, and (2) obesity suppresses the maturation of oligodendrocyte cells [19,20,21,22]. Furthermore, one recent study provided initial evidence for the relationship between obesity and lower myelin content in white matter regions [23]. However, the association between human obesity and intra-cortical myelination remains to be established to the best of our knowledge.

Research exploring the role of intra-cortical myelin on obesity remains limited, in large part due to challenges inherent in the quantification of intra-cortical myelin in vivo. Nevertheless, recent MRI studies have proposed the T1/T2-weighted signal ratio can be calculated as a reliable—albeit relative and indirect—in vivo indicator of intra-cortical myelin [24,25]. Myelin mapping derived from T1/T2 signal ratio has been shown to be consistent with histologically-derived maps of cortical myelin [26]. Specifically, the distribution of T1/T2 signal ratio reflects a well-known pattern of myelination in the cerebral cortex that emerges first in primary sensory-motor regions (e.g., motor cortex), followed by high order associative regions such as the temporal and parietal lobes (e.g., inferior parietal lobe), and eventually in the pre-frontal cortex (e.g., middle frontal gyrus) [27]. Also, the T1/T2 signal ratio has been applied to characterize intra-cortical myelin development across the lifespan [27,28]. Individual variation in the T1/T2 signal ratio has also been associated with individual variability in high order cognitive performances [28,29], sleep quality [30], and personality traits, such as neuroticism [31]. As such, these lines of research suggest that the T1/T2 signal ratio may be a valid indicator for elucidating the association between obesity and cortical myelination in human samples.

The aim of this study was to investigate the association between obesity and intra-cortical myelin, as measured by the T1/T2 ratio, within a large, socio-demographically-diverse young adult sample derived from the human connectome project database, 1200 Participants Release [32]. Given that intra-cortical myelin evolves during development and decays in aging [28], utilizing a young adult sample, i.e., with limited age range, can minimize the effect of age on the intra-cortical myelin, which would further help to obtain a relatively pure association between BMI and intra-cortical myelin. This database includes objective behavioral indexes and neuroimaging data with high-resolution and image quality, hence providing a unique opportunity to explore the important yet unresolved question about how obesity is related to intra-cortical myelin content. Considering the close relationship between intra-cortical myelin and neural activity [12,13,14,15], we expected obesity-related intra-cortical myelin alterations would provide a novel micro-structural basis for interpreting previously-documented neural activity abnormalities related to obesity. Based on the evidence reviewed above, we hypothesized that associations between obesity and intra-cortical myelin would be localized to brain regions that have been consistently engaged in reward processing (e.g., medial OFC), attention (e.g., visual cortex, middle temporal gyrus), and saliency detection (e.g., insula, supramarginal gyrus, and precuneus).

## 2. Materials and Methods

### 2.1. Participants

Participants included in this investigation were selected from the 1200 Subjects Release of the Human Connectome Project (HCP) from the Washington University–University of Minnesota (WU–Minn HCP) Consortium. This dataset provides high-quality behavioral/demographic and imaging data from healthy young adults [32] (https://www.humanconnectome.org/, accessed on 10 March 2021). The HCP database captures a broad range of variability in healthy young adults regarding behavioral, ethnic, and socioeconomic characteristics. Please refer to Van Essen et al. [32] for additional extensive details. From the original HCP dataset, we first included a group of participants (*n* = 1112) for which T1/T2-derived myelin maps as well as body mass index (BMI, calculated as self-reported weight in kilograms divided by height in meters squared) data were available. From this group, we further excluded data from 46 participants based on the exclusion criteria indicated as follows: (1) participants who were missing intermediate files for space transformation (see below for details); (2) participants with missing values on demographic variables such as age, sex, education, and race (*n* = 2) or family information (*n* = 3); (3) participants with a history of hyper/hypothyroidism (*n* = 4) or history of other endocrine problems (*n* = 16); and (4) women who had recently given birth (*n* = 4). As a result, the final sample comprised 1066 participants. Full informed consent from each participant was obtained by WU–Minn HCP Consortium, and research procedures and ethical guidelines were followed in compliance with WU institutional review board approval. This study was approved by the Human Ethics Committee at Southwest University (No. SWU20210305D), and conducted by following the guidelines of the Declaration of Helsinki.

### 2.2. MRI Scanning Protocol

T1 and T2 MRI data were collected with a Siemens 3T Tim Trios MRI scanner (12-channel head coil) at WU in St. Louis and Northwestern University. T1 and T2 scans were both acquired sagittally.

(1) T1 scans were performed using a 3D T1w magnetization-prepared rapid gradient echo (MPRAGE) sequence with the following scan parameters: TR = 2400 ms; TE = 2.14 ms; TI = 1000 ms; 8° flip angle; FOV = 224 × 224 mm; bandwidth = 210 Hz/pixel; echo spacing = 7.6 ms; voxel size = 0.7 mm × 0.7 mm × 0.7 mm; 256 slices; acquisition time = 7:40 min.
(2) T2 scans were performed using a 3D T2w sampling perfection with the application of an optimized contrast featuring a different angle evolution (SPACE) sequence with the following scan parameters: TR = 3200 ms; TE = 565 ms; variable flip angle; FOV = 224 × 224 mm; bandwidth = 744 Hz/pixel; echo spacing = 3.53 ms; voxel size = 0.7 mm × 0.7 mm × 0.7 mm; 256 slices; acquisition time = 8:24 min.

Additional extensive scanning details and parameters are provided in the WU–Minn HCP 1200 Subjects Data Release Reference Manual.

### 2.3. Image Processing

Preprocessed intra-cortical myelin maps were directly downloaded from the latest HCP consortium database (https://db.humanconnectome.org/, accessed on 10 March 2021). Such intra-cortical myelin maps were derived using T1-weighted and T2-weighted contrasts (refer to the description of the HCP consortium) [33], that can be found online (https://www.humanconnectome.org/storage/app/media/documentation/s1200/HCP_S1200_Release_Reference_Manual.pdf, accessed on 18 March 2021). The cortical myelin map was constructed in accordance with a standard, state-of-the-art HCP pipeline [34]. Briefly, this pipeline begins with the usage of the FreeSurfer software suite (version 5.2, http://surfer.nmr.mgh.harvard.edu/, accessed on 10 March 2021) to generate white, pial, and mid-thickness surfaces. These surfaces are then projected onto a 164-k vertex fs_LR mesh. During the preprocessing procedure, the T2w image was registered to the T1w image using FSL’s FLIRT algorithm and the mutual information cost function. Of note, the division of the T1w image by the aligned T2w image increases sensitivity to detect intra-cortical myelin and simultaneously decreases signal intensity biases. The contrast related to myelin content (m) is approximately proportional to the intensity in the T1-weighted image and approximately inversely proportional to the intensity (1/m) in the T2-weighted image, and the receive bias field can be represented by b in both images,. Hence, the generated contrast (T1w/T2w signal ratio) will be approximately proportional to m^2^, i.e.,

$$\frac{T1w}{T2w}\sim \frac{m*b}{\left(\frac{1}{m}\right)*b}=m^{2}$$

This means that we obtained an enhanced myelin contrast while mathematically eliminating most of the MR-related image intensity bias. In addition, given that T1-weighted and T2-weighted images are affected by uncorrelated noise, calculation of the T1/T2 ratio can result in an enhanced contrast-to-noise ratio for myelin.

### 2.4. Regional and Network Intra-Cortical Myelin Extraction

**Figure 1:**
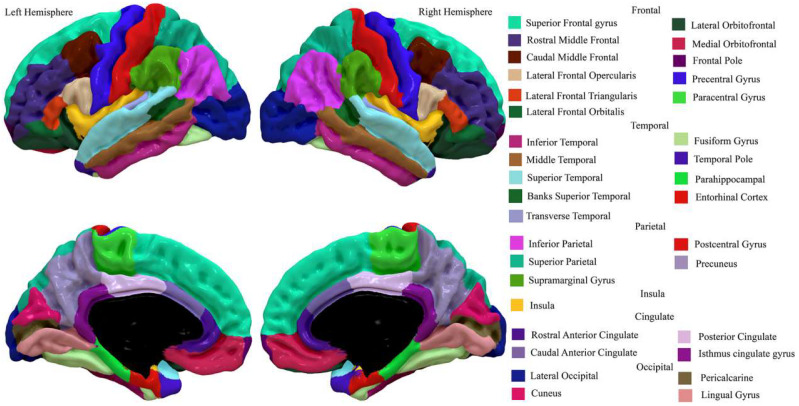
Regions in the DKT cortical labeling protocol.

First, we converted intra-cortical myelin maps for each participant to FreeSurfer fsaverage 164k vertex surface space using the Connectome workbench (https://www.humanconnectome.org/software/connectome-workbench, accessed on 18 March 2021). The FreeSurfer fsaverage surface space is a standard-subject, common space surface reconstruction template. Then, the Desikan–Killiany–Tourville atlas (DKT, [35]) was used to parcellate the cerebral cortex into 68 regions of interest (ROIs) for further analysis (Figure 1). DKT is a standard gyral-based neuroanatomical atlas that has been used widely in previous structural studies. Cortical myelin was averaged over all vertices in each ROI for each subject. Additionally, to increase generalizability of the findings at the brain network level, cortical myelin was also averaged over all vertices in each of seven network parcellations by Yeo et al. [36] for each subject. Yeo’s seven functional networks include visual, somatosensory, dorsal attention, ventral attention, limbic, frontoparietal, and default mode networks (Supplementary Materials Figure S1).

### 2.5. Statistical Analysis

For each region or network, the association between intra-cortical myelin measures and BMI was investigated using linear regression analyses. Because they are all known to affect brain structure and/or BMI [37,38,39,40,41,42,43,44], we also included age, sex, years of education, handedness, race (categorized as white or other), and evidence of drug consumption on test day as covariates of no interest in regression models. Considering the distribution of BMI was right-skewed, we used a log-transformation to obtain a normal-like BMI distribution. To take into account participants’ relatedness in the dataset, we used a permutation analysis of linear models (PALM) [45] to determine the significance for all association analyses in accordance with prior studies [46]. Importantly, permutation inference for the general linear model relies on only weak assumptions about the data, in which a null hypothesis could be tested simply by permuting the observations. Even in the presence of nuisance effects, non-independence, or apparent outliers in the data [47], permutation inference is powerful, while providing excellent type I error rate control in a wide range of imaging studies [48]. False discovery rate (FDR) correction was used to correct for multiple comparisons [49]. We considered an FDR corrected value of *p* < 0.05 to be significant. To visualize the results, significant regions were displayed using SurfIce (https://www.nitrc.org/projects/surfice/, accessed on 15 May 2021). To further obtain effect sizes for significant results, partial correlation coefficients were directly derived from the general linear model fits.

Considering total intracranial volume (TIV), substance use (i.e., alcohol use, tobacco use, marijuana use), and fluid intelligence (Table 1) might individually affect myelination and/or obesity, we conducted additional, exploratory control analyses to investigate potential effects of these variables on intra-cortical myelin-BMI association. Specifically, TIV, substance use, and fluid intelligence were separately included with the above six basic covariates (i.e., age, sex, years of education, handedness, race, and evidence of drug consumption on test day) in a linear regression model. We also ran an analysis including all covariates in a linear regression model.

## 3. Results

### 3.1. Participants’ Demographics

Demographics and other behavioral characteristics of the final sample are summarized in Table 1. The sample comprised 498 men and 568 women, had a mean age of 28.79 ± 3.71 years with a range from 22 to 37, was predominantly Caucasian (74.6%), and had a mean BMI of 26.44 ± 5.13 kg/m^2^ (range: 16.48 to 47.76). Following established BMI cutoffs, the cohort consisted of 478 lean participants (BMI < 25), 361 overweight participants (25 ≤ BMI < 30), and 227 participants with obesity (BMI ≥ 30). Table 1 provides more details regarding participant demographics.

### 3.2. BMI Relates to Abnormalities of Regional Intra-Cortical Myelin

**Figure 2:**
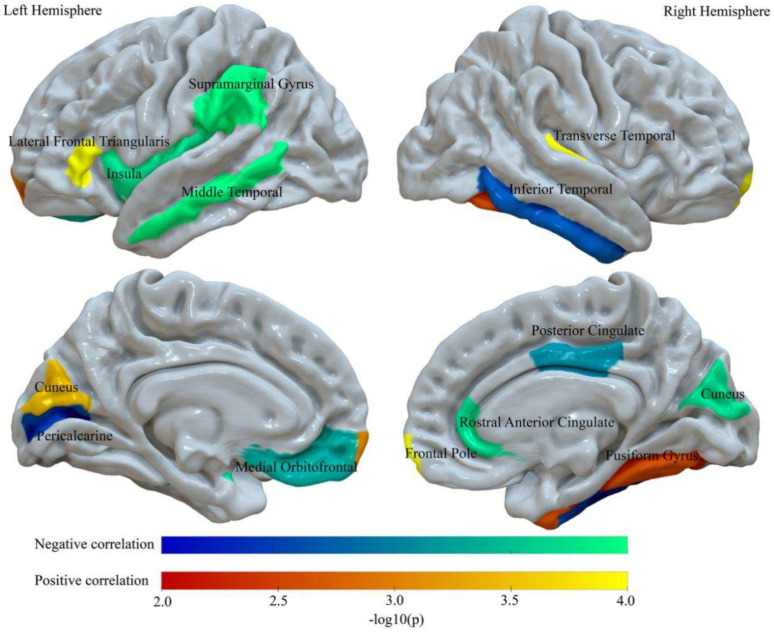
Regions showing a significant correlation between BMI and intra-cortical myelin. The warm color represents a significant positive correlation between BMI and intra-cortical myelin. The cold color represents a significant negative correlation between BMI and intra-cortical myelin. All results are shown after FDR correction (*p* < 0.05).

First, we investigated relations between individual BMIs and regional intra-cortical myelin content using the T1/T2 MRI signal ratio. There were 15 significant associations between BMI and regional intra-cortical myelin after performing permutation-based FDR correction (*p* < 0.05, corrected). All regions correlated with BMI are shown in Figure 2. Statistical values of all implicated regions are shown in Table 2. Nine regions had significant negative correlations. reflecting higher intra-cortical myelin levels corresponding to lower BMI values, including the left medial orbitofrontal gyrus; insula; supramarginal gyrus; middle temporal gyrus; pericalcarine cortex and right cuneus; rostral anterior cingulate gyrus; posterior cingulate gyrus; and inferior temporal gyrus. In addition, six regions had significant positive correlations between intra-cortical myelin and BMI, including the left lateral frontal triangularis; frontal pole and cuneus; right frontal pole; fusiform gyrus; and transverse temporal gyrus. Notably, covariate analyses indicated that, even after controlling for individual differences in total intracranial volume, substance use, and fluid intelligence, previously observed intra-cortical myelin-BMI associations remained statistically significant (Supplementary Materials Figures S2–S5). To further test for the specificity of BMI-related effects, we repeated the analyses by including Hemoglobin A1C (HbA1c, Table 1) as a covariate in a linear regression model. Overall, we found that most of the associations between BMI and intra-cortical myelin observed without adjusting for HbA1c remained significant when adjusting for HbA1c, except that right inferior temporal gyrus were not significantly related to BMI (please see Supplementary Materials Table S1). In addition, we tested whether the associations between BMI and intra-cortical myelin differed between male and female participants for each distinct ROI. There was no region surviving after FDR correction (*p* > 0.05), suggesting that sex has no significant influence on the associations between BMI and intra-cortical myelin.

### 3.3. Associations between BMI and Network-Level Intra-Cortical Myelin Abnormalities

**Figure 3:**
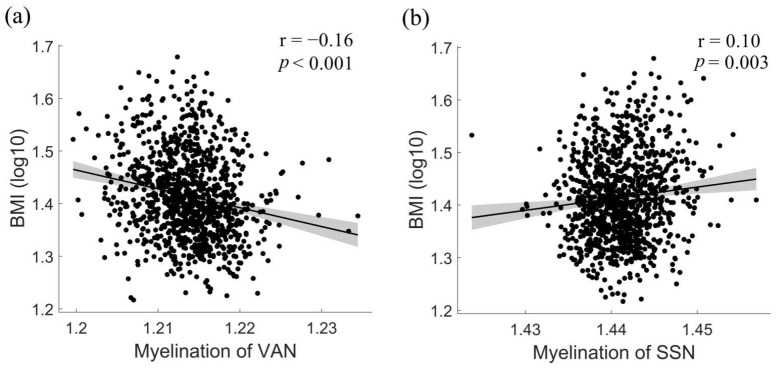
BMI relations to abnormalities in network-level intra-cortical myelin. (**a**) Negative correlation between BMI and intra-cortical myelin within the VAN. (**b**) Positive correlation BMI and intra-cortical myelin within the SSN. VAN, ventral attention network; SSN, somatosensory network.

Next, we investigated relations between individual differences in BMI and intra-cortical myelin content at a systems-level of brain functional networks, i.e., Yeo’s seven networks (Figure 3a). We found a significant negative association between BMI and intra-cortical myelin in the ventral attentional network (VAN, F = 23.441, Adjusted R^2^ = 0.129, Beta = 0.156, partial r_SSN = 0.160, permutation *p* = 0.003, Figure 3) and a significant positive association between BMI and intra-cortical myelin content in the somatosensory network (SSN, F = 5.358, Adjusted R^2^ = 0.028, Beta = 0.099, partial r_SSN =0.10, permutation *p* = 0.003). Critically, each of these associations was statistically significant, even after controlling for demographics, total intracranial volume, reported substance use, and fluid intelligence; hence covariates did not significantly affect these two intra-cortical myelin-BMI associations (TIV as a covariate of no interest, F_SSN = 4.864, Adjusted R^2^_SSN = 0.028, Beta_SSN = 0.101, partial r_SSN = 0.10, permutation *p* = 0.006; F_VAN = 20.537, Adjusted R^2^_VAN = 0.128, Beta_VAN = 0.155, partial r_VAN = 0.16, permutation *p* < 0.001; substance use as a covariate of no interest, F_SSN = 3.458, Adjusted R^2^_SSN = 0.024, Beta_SSN = 0.097, partial r_SSN = 0.09, permutation *p* = 0.002; F_VAN = 17.062, Adjusted R^2^_VAN = 0.137, Beta_VAN = 0.169, partial r_VAN = 0.17, permutation *p* < 0.001; fluid intelligence as a covariate of no interest, F_SSN = 4.816, Adjusted R^2^_SSN = 0.028, Beta_SSN = 0.098, partial r_SSN = 0.09, permutation *p* = 0.002; F_VAN = 20.690, Adjusted R^2^_VAN = 0.130, Beta_VAN = 0.156, partial r_VAN = 0.15, permutation *p* < 0.001; all 11 background variables as covariates of no interest, F_SSN = 3.103, Adjusted R^2^_SSN = 0.024, Beta_SSN = 0.104, partial r_SSN = 0.10, permutation *p* = 0.003; F_VAN = 14.191, Adjusted R^2^_VAN = 0.136, Beta_VAN = 0.167, partial r_VAN = 0.16, permutation *p* < 0.001).

## 4. Discussion

This research represents the first evidence that obesity is associated with an aberrant pattern of intra-cortical myelin within a large, socio-demographically diverse sample of healthy young adults (*n* = 1066). Specifically, as hypothesized, an obese weight status, as measured by BMI, was related to attenuated intra-cortical myelin content in regions involved in reward processing (i.e., medial orbitofrontal cortex, rostral anterior cingulate cortex), attention (i.e., visual cortex, inferior/middle temporal gyrus, posterior cingulate cortex), and salience detection (i.e., insula, supramarginal gyrus), in addition to having positive correlations with intra-cortical myelin content in regions associated with somatosensory processing (i.e., somatosensory network) and inhibitory control (i.e., lateral inferior frontal gyrus, frontal pole). These associations were statistically significant, even after adjusting for total intracranial volume, reported substance use, fluid intelligence, and participant demographics. Taken together, abnormal intra-cortical myelin may represent a novel micro-structural substrate underlying previously observed associations between obesity and brain neural activity during exposure to visual food cues [3,4,5,6,7]. As such, the results may inform current neurobiological theories of obesity [50,51,52] that have emphasized the importance of enhanced incentive salience of food cues in determining susceptibility to obesity.

### 4.1. Lower Intra-Cortical Myelin in Regions Involved in Reward Processing, Attention, and Salience Detection

Theory and experimental research suggest that obesity is related to enhanced incentive salience of food cues [50,51,52]. For example, neuroimaging studies have consistently observed obesity is associated with stronger food cue-induced activation in regions implicated in reward processing and valuation encoding during exposure to food imagery, i.e., the OFC and rACC [3,4,5,6,7,53] as well as regions involved in attention toward and salience detection of food-related visual reward cues, i.e., occipital lobe, inferior and middle temporal gyrus, insula, supramarginal gyrus, and posterior cingulate [6,7,8,9,10,54,55]. Notably, we found higher BMIs were related to lower intra-cortical myelin levels in most of these regions. Recent evidence suggests that intra-cortical myelin fosters cognitive functioning in the brain, potentially by facilitating local neural synchronization [16]. Similarly, the biological significance of demyelination is thought to reflect associated processes including conduction failures and spike-time arrival delays [13,56]. Although speculative, a hypothesis that follows from this pattern of relations is that obesity-related reductions in intra-cortical myelin decrease nerve conduction velocities and neural signal integrity in these regions, hence contributing to abnormal reward processing, attention, and salience detection during exposure to food cues.

We also found participants with higher BMIs tended to display relative deficits in intra-cortical myelin within the ventral attention network (VAN). This network is also referred to as ae salience network in the literature [57], and is thought to be a core neural system involved in enabling the dynamic integration of homeostatic (internal) processes with salience (external) information, thus helping organisms to perceive and respond appropriately to changing environmental conditions [58]. Research has demonstrated that people with obesity show abnormal activation in core structures (insula and ACC) of the salience network during exposure to visual food stimuli [59,60]. Notably, too, at rest, people with higher BMIs can show abnormal intrinsic connectivity within the salience network [61,62,63]. Our findings extend previous work by showing how intra-cortical myelin of the salience network is reduced among young adults with higher BMIs. Other recent work has identified patterns of distributed brain regions sharing similar myeloarchitecture that correspond to underlying functional connectivity [64]. Hence, we speculate that different levels of intra-cortical myelin within the VAN may underlie the functional integrity of this network. In tandem with previous research [61], our findings suggest that abnormal intra-cortical myelination within the salience network may contribute to overeating and/or reduced energy expenditure in obesity through creating imbalances between autonomic homeostatic processing and salient reward processing of visual food cues.

### 4.2. Enhanced Intra-Cortical Myelin in Regions Involved in Somatosensory Processing and Inhibitory Control

In contrast to these deficits in intra-cortical myelin, we also found people with higher BMIs displayed more intra-cortical myelination in the somatosensory network. Previous fMRI studies have found that elevated reward circuitry responsivity in people with obesity can be accompanied by additional recruitment of increased somatosensory region responsiveness when viewing food cues [4,6]. One intriguing proposal offered by these authors is that additional recruitment of sensorimotor cortices associated with eating in people with obesity reflects heightened anticipation or mental stimulation of eating behavior in response to viewing food cues that reinforces well-established, automatic unhealthy eating habits that can perpetuate obesity [6]. In addition, a recent study found that, at rest, participants with higher BMIs tended to display increased integration in the somatosensory network, further suggesting increased sensory-driven behavior as a possible mechanism underpinning overeating and subsequent weight gain [65]. It is notable that sensory-motor cortices have the highest levels of myelin and earliest myelination during brain maturation [66,67,68]. The observed positive correlation between somatosensory network myelin and BMI suggests that signal propagation and neural signal integrity within the somatosensory network among people with higher BMIs enhances speed in automatic processing of food cues, in a manner that reinforces unhealthy eating habits and/or hampers the capacity to inhibit responses to food cues and lose weight later in life.

In addition, BMI was positively correlated with intra-cortical myelin in the frontal pole and lateral inferior frontal gyrus. The frontal pole subserves the monitoring of outcomes expected from ongoing courses of action, while the inferior frontal gyrus is relevant to inhibition and attentional control [69]. Some studies have suggested that evaluated responses to food cues in brain regions linked to reward may drive overeating among people with obesity when such responses are coupled with insufficient inhibitory control [70]. In humans, the myelin of associative regions, including the prefrontal and temporo-parietal cortices, tends to maturate later throughout the lifespan compared to sensory-motor regions and consequently show light myelin content [66,67,68]. This important developmental difference reflects a more complex cyto-architecture of associative regions in terms of underlying micro-structures [66,67,68]. One hypothesis that follows from such data is that positive relations of BMI with myelin content in the prefrontal pole may result from premature development of the prefrontal pole that is expected to have relatively light myelin content and prolonged myelination, that might lead to insufficient development of inhibitory control capacities in frontal lobe regions. Longitudinal designs and histological investigations are needed to test this contention and elucidate mechanisms underlying the increased intra-cortical myelin in frontal regions among people with higher BMIs.

### 4.3. Implications

Findings of this study provided new insights into the association between BMI and brain health, by highlighting a micro-structural substrate underlying BMI-related neural activity. Broadly speaking, this evidence can be merged with accumulating evidence for the profound impact of diet and nutrition on brain health. Our findings implied that it is important to maintain an appropriate BMI status. Therefore, strategies to prevent obesity and help people with higher BMIs to lose weight and prevent weight regain should be encouraged; these include healthy eating and regular physical activity. Because neuroimaging studies have consistently observed that obesity is associated with stronger food cue-induced activation in regions implicated in reward processing during exposure to food imagery, government policies to reduce exposure to food advertisements and advocate for healthy dietary pattern also warrant consideration.

### 4.4. Strength and Limitations

A key strength of our study was its use of a large young adult sample which facilitated the identification of reliable and potentially subtle links of BMI with intra-cortical myelin. In addition, analyses underscored the stability of significant associations, even after statistically controlling for several key potential confounding factors in participant backgrounds. Nevertheless, the main limitations of this study should also be acknowledged. First, due to the cross-sectional study design, directions of causality between BMI and intra-cortical myelin could not be clarified. Longitudinal extensions and intervention studies are needed to clarify causal directions of such relations. In addition, we must acknowledge that, although self-reported BMI is widely used in neuroimaging studies, biases in self-reported BMI data do exist. Moreover, although the HCP dataset is based upon a state-of-art data processing pipeline, possible errors may have resulted in an imperfect surface reconstruction that influenced myelin content estimates. Signal intensity was averaged across each ROI to obtain an average measure per region [29], and spatial acuity that may be pertinent to detecting potentially subtle intra-regional differences associated with obesity. Third, even though BMI is a widely adopted proxy measure of obesity status, it is a relatively crude measure of body density that does not consider relevant physical attributes such as muscle mass and anthropometric features. Therefore, future research should complement this work with more direct measures of adiposity, such as body fat. In addition, because our sample comprised of young adults, results may not apply to BMI-myelin relations during other developmental phases such as childhood, middle age, and old age. Finally, despite controlling for several key background factors in the analyses, the possible impact of other, unmeasured factors, including genetics, personality, and lifestyle factors on associations cannot be dismissed.

## 5. Conclusions

In conclusion, drawing from a large, demographically diverse young adult sample, we demonstrated for the first time that obesity has significant associations with intra-cortical myelin in key regions of interest implicated in neurobiological explanations of obesity. Specifically, obesity was related to lower intra-cortical myelin content in regions involved in reward processing, attention, and salience detection as well as increased intra-cortical myelin content in regions associated with somatosensory processing and inhibitory control. As such, results provide foundations for the hypothesis that altered intra-cortical myelin represents a novel micro-structural substrate underlying abnormal obesity-related neural activity Future investigations should also evaluate effects of genetics and lifestyle factors, such as diet and physical activity, on intra-cortical myelination.

## Figures and Tables

**Table 1:** Demographic characteristics of participants ^a^.

Variable	
Body Mass Index (BMI), mean (SD), Kg/M^2^	26.44 (5.13)
Age, mean (SD), years	28.79 (3.71)
Male (Sex), N (%)	498 (46.72)
Handedness, mean (SD) ^b^	66.03 (43.82)
Education, mean (SD), years	14.92 (1.80)
Race N (%)	
White	796 (74.67)
Other	270 (25.33)
Evidence of drug consumption on test day, N (%)	140 (13.13)
Fluid Intelligence, mean (SD) ^c^	16.88 (4.80)
Total brain volume, mean (SD), mm^3^	1582972.87
Smoking history, N (%)	
Never	565 (53.00)
Smoked 1–19 cigarettes over lifetime	92 (8.63)
Smoked ≥20 cigarettes over lifetime	126 (11.82)
Regular smoker	283 (26.55)
Substance use	
Frequency of alcohol use in the last 12 months, mean (SD) ^d^	4.27 (1.56)
Marijuana use, mean (SD), incidents	1.41 (1.69)
Hemoglobin A1C (HbA1c), mean (SD) ^e^	5.23% (0.30)

^a^ Further definitions are available at the Human Connectome Project Data Dictionary. ^b^ Handedness of participants from −100 to 100 is assessed using the Edinburgh Handedness questionnaire. Negative numbers indicate that a participant is more left-handed than right-handed, while positive numbers indicate that a participant is more right-handed than left-handed. ^c^ Fluid intelligence was measured by number of correct responses during the penn matrix test. This test measures fluid intelligence via non-verbal reasoning using an abbreviated version of the Raven’s Progressive Matrices Form A developed by Gur and colleagues [32]. These data are available for 1058 participants. ^d^ Frequency of any alcohol use in the past 12 months was assessed on an inverse scale of 1 to 7, where alcohol use on 4–7 days per week was scored 1 for male participants and 2 for female participants; 3 days/week was scored 2; 2 days/week was scored 3; 1 day/week was scored 4; 1–3 days/month was scored 5; 1–11days/year was scored 6; and no use in the past 12 months was scored 7 for both sexes. These data are available for 1012 participants. ^e^ These data are available for 735 participants.

**Table 2:** Regions showing significant correlations between BMI and intra-cortical myelin.

Region	F	Adj. R^2^	Beta	B	CI of B(95%)	DW	Partial r Value	Permutation *p* Value(−log10)
**Negative Correlations**								
LMOFG	11.08	0.062	−0.10	−0.24	(−0.39,−0.10)	1.97	−0.097	0.0005 (3.30)
L MTG	12.33	0.069	−0.13	−1.59	(−2.33,−0.87)	1.95	−0.129	0.0001 (4)
L Perical	10.64	0.060	−0.08	−0.39	(−0.68,−0.11)	1.95	−0.082	0.0028 (2.55)
L SMG	13.87	0.078	−0.16	−2.26	(−3.09,−1.43)	1.93	−0.159	0.0001 (4)
L Insula	11.87	0.073	−0.12	−0.68	(−1.02,−0.34)	1.95	−0.113	0.0002 (3.70)
R Cuneus	12.41	0.07	−0.13	−0.73	(−1.06,−0.40)	1.96	−0.132	0.0002 (3.70)
R ITG	10.99	0.062	−0.09	−1.11	(−1.81,−0.41)	1.95	−0.095	0.0021 (2.68)
R PCG	11.31	0.064	−0.11	−0.74	(−1.15,−0.37)	1.94	−0.106	0.0008 (3.10)
R rACG	11.77	0.066	−0.12	−0.76	(−1.15,−0.37)	1.94	−0.115	0.0002 (3.70)
**Positive correlations**								
L Cuneus	11.64	0.065	0.11	0.79	(0.38,1.21)	1.94	0.113	0.0004 (3.40)
L lFT	12.31	0.069	0.13	1.02	(0.55,1.49)	1.95	0.129	0.0001 (4)
L FP	11.24	0.063	0.10	0.29	(0.12,0.46)	1.94	0.102	0.0010 (3)
R FG	10.73	0.06	0.09	1.11	(0.34,1.88)	1.95	0.085	0.0026 (2.59)
R FP	13.80	0.078	0.16	0.47	(0.30,0.65)	1.93	0.156	0.0001 (4)
R TTG	13.05	0.073	0.15	0.35	(0.21,0.49)	1.94	0.143	0.0001 (4)

F, F value on the F distribution; B, unstandardized coefficients; CI, Confidence interval; DW, Durbin-Watson; Negative Correlations, negative correlations between BMI and intra-cortical myelin; L, left hemisphere; R, right hemisphere; MOFG, Medial Orbitofrontal Gyrus; MTG, Middle Temporal Gyrus; Perical, Pericalcarine cortex; SMG, Supramarginal Gyrus; ITG, Inferior Temporal Gyrus; PCG, Posterior Cingulate Gyrus; rACG, Rostral Anterior Cingulate Gyrus; lFT, Lateral Frontal Triangularis; Positive correlations, positive correlations between BMI and intra-cortical myelin; FP, Frontal Pole; FG, Fusiform Gyrus; TTG, Transverse Temporal Gyrus.

## Data Availability

Data were downloaded from the HCP consortium database (https://db.humanconnectome.org/, accessed on 10 March 2021).

## Supplementary Materials

The following are available online at https://www.mdpi.com/article/10.3390/nu13093221/s1, Figure S1: Yeo brain network classification. Figure S2: The confidence intervals of all significant regions after adjusting for total intracranial volume. Figure S3: The confidence intervals of all significant regions after adjusting for substance use. Figure S4: The confidence intervals of all significant regions after adjusting for fluid intelligence. Figure S5: The confidence intervals of all significant regions after adjusting for all covariates. Table S1: Regions showing significant correlations between BMI and intra-cortical myelin after adjusting for HbA1c.
