# Supplementary material for: The Association between Body Mass Index and Intra-Cortical Myelin: Findings from the Human Connectome Project
Source: Nutrients. 2021 Sep 16;13(9):3221. doi: 10.3390/nu13093221 (PMC8469469; doi:10.3390/nu13093221)
Supplement: Supplementary file 1 [file nutrients-13-03221-s001.zip › nutrients-1339162-supplementary.pdf]

Supplementary Table S1

Regions showing significant correlations between BMI and intra-cortical myelin after adjusting for HbA1c

**Region/Network**	**partial r value**	**Permutation p value(−log10)**
**Negative correlations**		
SSN	0.0660	0.0488
LMOFG	0.0887	0.0113
L MTG	0.0839	0.0227
L Perical	0.0707	0.0424
L SMG	0.1185	0.0008
L Insula	0.1085	0.0007
R Cuneus	0.1228	0.0003
R ITG	0.045	0.1384
R PCG	0.1027	0.004
R rACG	0.1163	0.0006
**Positive correlations**		
VAN	0.1217	0.0002
L Cuneus	0.1123	0.0024
L lFT	0.1491	0.0001
L FP	0.1265	0.0004
R FG	0.0698	0.0302
R FP	0.1772	0.0001
R TTG	0.1025	0.0019

L, left hemisphere; R, right hemisphere; SSN, somatosensory network; MOFG, Medial Orbitofrontal Gyrus; MTG, Middle Temporal Gyrus; Perical, Pericalcarine cortex; SMG, Supramarginal Gyrus; ITG, Inferior Temporal Gyrus; PCG, Posterior Cingulate Gyrus; rACG, Rostral Anterior Cingulate Gyrus; lFT, Lateral Frontal Triangularis; FP, Frontal Pole; FG, Fusiform Gyrus; TTG, Transverse Temporal Gyrus; VAN, ventral attention network.

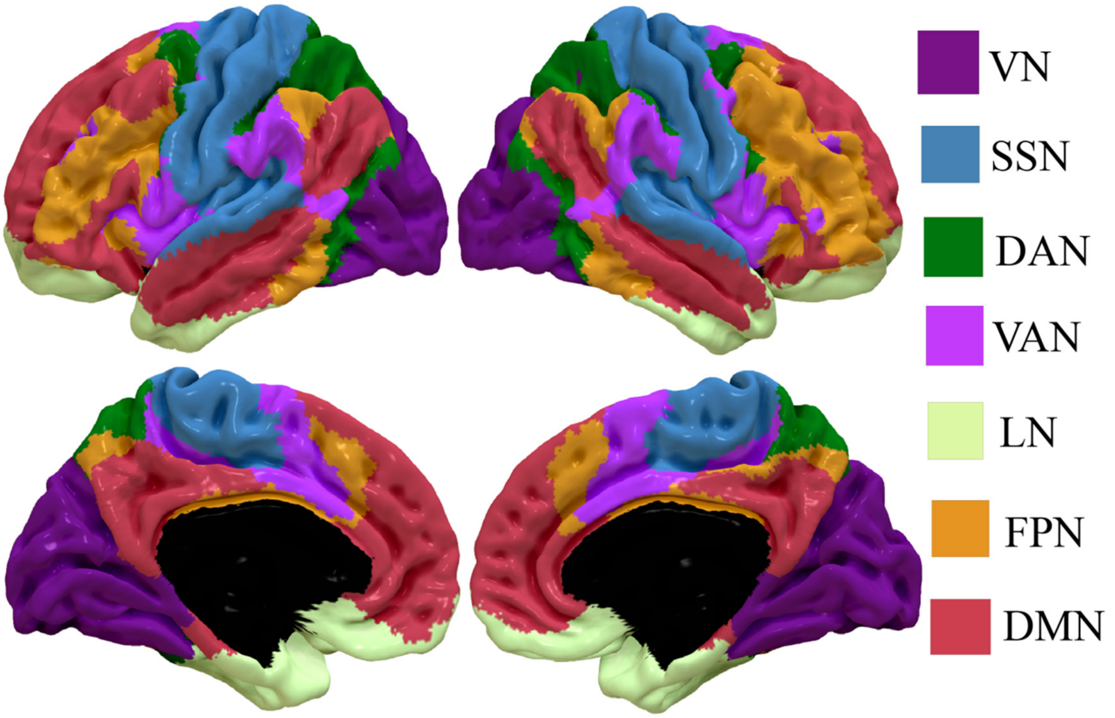

Supplementary Figure S1. Yeo brain network classification [1]. VN, visual network; SSN, somatosensory network; DAN, dorsal attention network; VAN, ventral attention network; LN, limbic network; FPN, frontal-parietal network; DMN, default mode network;

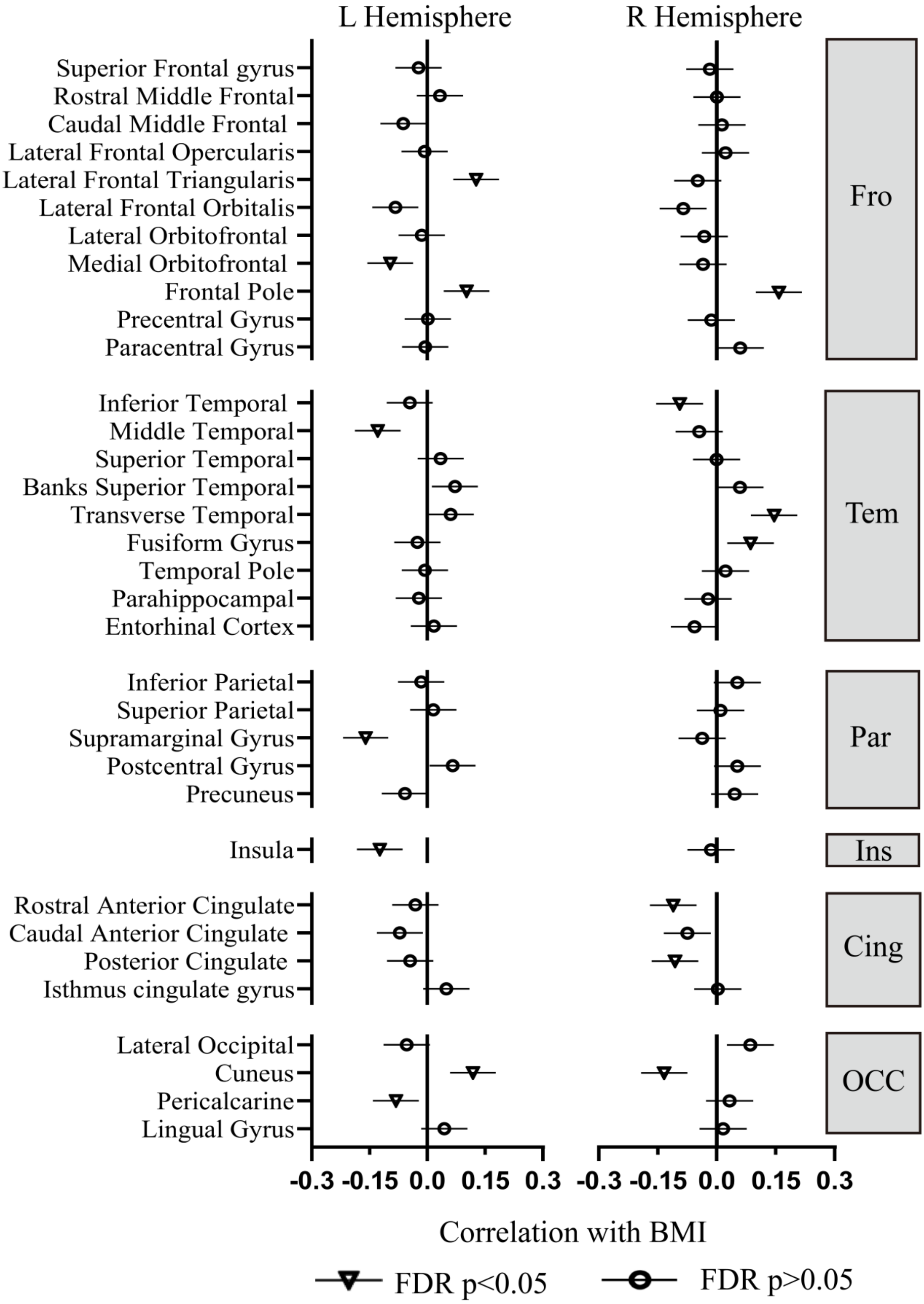

Supplementary Figure S2. The confidence intervals of all significant regions after adjusting for total intracranial volume. The error bar represents the 95% confidence intervals. Fro, frontal lobe; Tem, temporal lobe; Par, parietal lobe; Ins, insula; Cing, cingulate; OCC, occipital lobe.

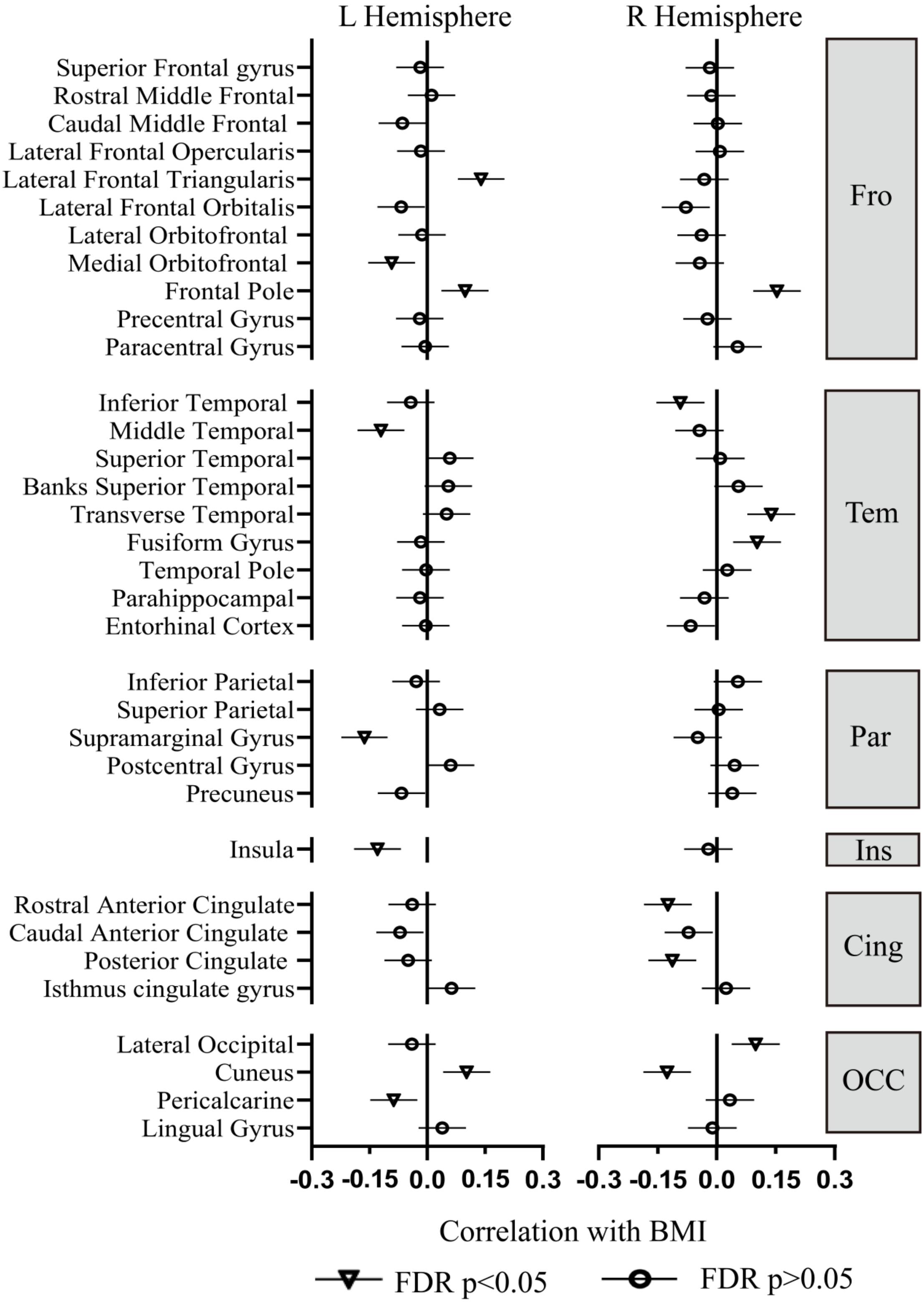

Supplementary Figure S3. The confidence intervals of all significant regions after adjusting for substance use. The error bar represents the 95% confidence intervals. Fro, frontal lobe; Tem, temporal lobe; Par, parietal lobe; Ins, insula; Cing, cingulate; OCC, occipital lobe.

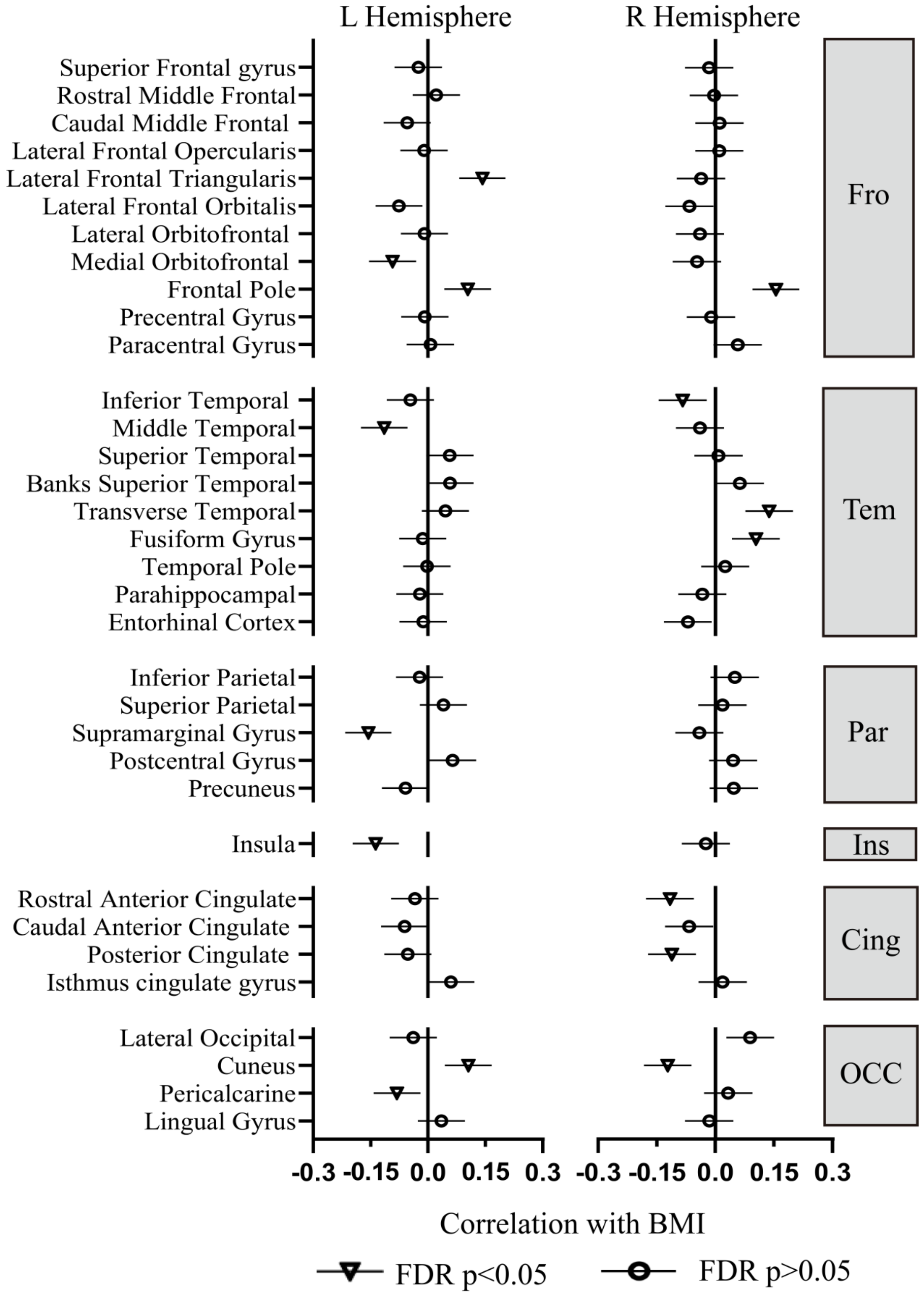

Supplementary Figure S4. The confidence intervals of all significant regions after adjusting for fluid intelligence. The error bar represents the 95% confidence intervals. Fro, frontal lobe; Tem, temporal lobe; Par, parietal lobe; Ins, insula; Cing, cingulate; OCC, occipital lobe.

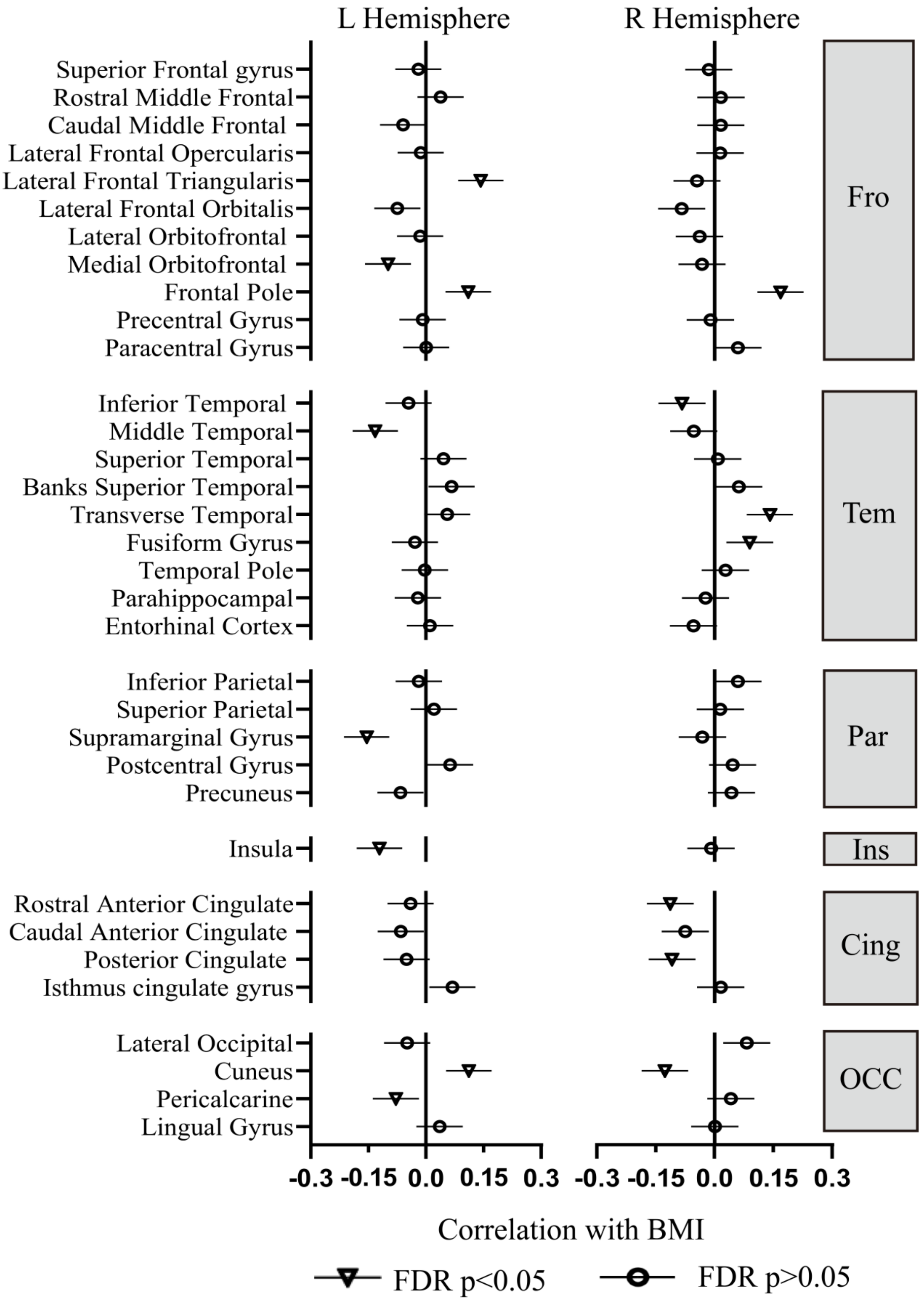

Supplementary Figure S5. The confidence intervals of all significant regions after adjusting for all covariates. The error bar represents the 95% confidence intervals. Fro, frontal lobe; Tem, temporal lobe; Par, parietal lobe; Ins, insula; Cing, cingulate; OCC, occipital lobe.

References in this material

1. Yeo, B.T.; Krienen, F.M.; Sepulcre, J.; Sabuncu, M.R.; Lashkari, D.; Hollinshead, M.; Roffman, J.L.; Smoller, J.W.; Zöllei, L.; Polimeni, J.R. The organization of the human cerebral cortex estimated by intrinsic functional connectivity. *J. Neurophysiol.* **2011**, 106, 1125–1165.
